# Prediction of the Ultimate Strength of Notched and Unnotched IM7/977-3 Laminated Composites Using a Micromechanics Approach

**DOI:** 10.3390/polym13203491

**Published:** 2021-10-11

**Authors:** Manzar Masud, Moosa S. M. Al Kharusi, Muhammad Umair Ali, Aamir Mubashar, Shaik Javeed Hussain, Adnan Tariq, Gulfam Ul Rehman, Mahmood Hassan Akhtar, Shama Javeed

**Affiliations:** 1 Computational Mechanics Group, Department of Mechanical Engineering, School of Mechanical and Manufacturing Engineering (SMME), National University of Sciences and Technology (NUST), H-12, Islamabad 44000, Pakistan; manzarmasud.pg@smme.edu.pk; 2 Department of Mechanical Engineering, Capital University of Science and Technology (CUST), Islamabad 44000, Pakistan; 3 Department of Mechanical and Industrial Engineering, College of Engineering, Sultan Qaboos University, 123 Al Khoud, Muscat 112, Oman; 4 Department of Unmanned Vehicle Engineering, Sejong University, Seoul 05006, Korea; umair@sejong.ac.kr; 5 Department of Electrical and Electronics, Global College of Engineering and Technology, Muscat 112, Oman; 6 Department of Mechanical Engineering, University of Wah, Wah Cantt 47040, Pakistan; adnan.tariq@wecuw.edu.pk (A.T.); gulfam.rehman@wecuw.edu.pk (G.U.R.); 7 Department of Chemistry, National University of Technology (NUTech), Islamabad 44000, Pakistan; mahmoodhassan@nutech.edu.pk; 8 Department of Mathematics, Pusan National University, Busan 46241, Korea; shamajaveed@pusan.ac.kr

**Keywords:** multiscale simulation, micromechanics of failure, ultimate strength, IM7/977-3, composite laminates

## Abstract

This paper proposes a multi-scale analysis technique based on the micromechanics of failure (MMF) to predict and investigate the damage progression and ultimate strength at failure of laminated composites. A lamina’s representative volume element (RVE) is developed to predict and calculate constituent stresses. Damages that occurred in the constituents are calculated using separate failure criteria for both fiber and matrix. Subsequently, the volume-based damage homogenization technique is utilized to prevent the localization of damage throughout the total matrix zone. The proposed multiscale analysis procedure is then used to investigate the notched and unnotched behavior of three multi-directional composite layups, [30, 60, 90, −60, 30]_2S_, [0, 45, 90, −45]_2S_, and [60, 0, −60]_3S_, subjected to static tension and compression loading. The specimen is fabricated from unidirectionally reinforced composite (IM7/977-3). The prediction of ultimate strength at failure and equivalent stiffness are then benchmarked against the experimental test data. The comparative analysis with various failure models is also carried out to validate the proposed model. MMF demonstrated the capability to correctly predict the ultimate strength at failure for a range of multidirectional composites laminates under tensile and compressive load. The numerically predicted findings revealed a good agreement with the experimental test data. Out of the three investigated composite layups, the simulated results for the quasi-isotropic [0, 45, 90, −45]_2S_ layup agreed extremely well with the experimental results with all the percentage errors within 10% of the measured failure loads.

## 1. Introduction

Composite laminate-based structures are extensively used in aerospace applications [1]. They are characterized as a significant configuration of composites that are critical for different categories of exceedingly loaded structures. Due to the elevated strength–weight ratio, high specific modulus, and the ability to be customized for a particular application, composite materials offer a variety of advantages compared to other conventional materials [2]. In view of the recent developments in the advanced aerospace industry and progressively high demand for increased performance of laminated composites structures, more sophisticated design and failure prediction models are required [3]. 

Reliable failure theories and progressive damage models are needed to precisely predict the complex failure phenomenon in the structures made of composites. Therefore, predictive tools that require a reduced number of essentials tests are becoming more important because of the extremely expensive tests on composite structures [4]. Computational multi-scale modelling and simulation tools for the prediction of damage mechanisms, progressive damage, and residual strengths can be used to achieve this ambitious goal [5,6]. However, the concerns like an-isotropy, non-linear stress–strain response, complex failure processes contribute to the difficulty of modeling the structural and damage behavior of composites, especially the failure’s initiation and propagation, subsequently leading to the prediction of ultimate strength [7,8].

Progressive Damage Analysis (PDA) and ultimate strength at failure’s prediction in composite laminates is a difficult task, even after many years of publication of ground-breaking works on failure theories by Rosen [9] and Tsai and Wu [10]. Even with composite structures subjected to constant in-plane loading, ultimate strength prediction has shown serious issues, as mentioned in different World-Wide Failure exercises [11,12]. The main problems and intricacy lie in the interaction and mixture of various damage modes in laminates, such as fiber-failure, matrix-cracking, and delamination, which ultimately result in residual strength and structural integrity loss. 

Progressive failure simulation and modeling approaches focus on the growth of damage and the interaction of different phenomena in composite laminates. To evaluate the progressive damage approaches, a benchmarking exercise [13] organized by “Air Force Research Laboratory” (AFRL) [13], titled “Damage Tolerance Design Principles (DTDP),” was arranged to investigate the capability of different PDA models against AFRL provided test data. The main aim of the exercise was to predict the ultimate strength of several IM7/977-3 carbon fiber reinforced laminates under tensile and compressive loading. During this study, nine different analysis teams forecasted the ultimate strength of the unnotched and open-hole specimen. These predictions were made for three multidirectional laminate groups [14], which are [30, 60, 90, −60, −30]_2S_, [0, 45, 90, −45]_2S_ and [0, 60, 60]_3S_.

The analysis codes presented in the DTDP exercise can be divided into two categories depending upon the type of methodology, i.e., macromechanics and micromechanics [13]. From the presented analysis codes, the b-spline analysis method with mesh independent cracking (BSAM with MIC) [13] and discrete crack network (DCN) [15] was based on the macromechanics level. In macromechanics, damage in the matrix and fibers is computed using composite level macro stresses. In contrast, the micromechanics approach utilizes stress and strain information from each of the composite constituents. 

Using the micromechanics approach to perform the PDA of composite laminates is of great significance because of non-linearity before final failure, which most first ply failure criteria cannot capture. Furthermore, the micromechanics approach facilitates accounting precisely for changes in the properties of constituents in composite materials and the impact of the microstructure, such as the fiber-orientation and fiber volume ratio, which makes it a powerful tool to estimate progressive damage in composite based structures. 

In the DTDP exercise [13], the analysis codes, namely generalized method of cells (MAC/GMC) [16], helius progressive failure analysis (Helius PFA) [17], enhanced schapery theory (EST) [13], multi-scale design system for linking continuum scales (MDS-C) [18], general optimization analyzer (GENOA) [19], eigendeformation-based reduced order homogenization (EHM) [20], and n-phase cylindrical model (NCYL) [21], were based on the micromechanics approach.

The failure theories based on micromechanics have become increasingly attractive for researchers in modeling the failure patterns of composite-based structures due to the benefit in describing the failure processes at the constituent level [22]. By combining the representative unit cells of the meso level and micro level, the multiscale analysis technique takes both the accuracy of micromechanics-based failure technique and efficiency of macro-based analysis. Gosse et al. [23] suggested a theory based on the stress invariant to predict the commencement of damage in composites by utilizing the strain-based enhancement factors. 

A micromechanics-based modeling technique using stress invariants was proposed by Tran et al. [24]. Mayes et al. [25] suggested micromechanics depended on multi-continuum theory (MCT), in which the stress and strain fields are obtained using a FE analysis. Ha et al. [26] proposed a damage model based upon the micromechanics of failure (MMF) technique for the estimation of failure in composite material’s constituents using stress amplification factors. MMF is a constituent-based progressive damage model in which the data is transferred from the microscale model to the mesoscale model and vice versa. Lei et al. utilized MMF to accurately forecast the ultimate failure strength of woven configuration [27] and braided configuration [28] textile composites. 

Considering the advantage of MMF in prediction accuracy, the MMF model is utilized to estimate the failure strength and stiffness of coupons fabricated from unidirectionally reinforced composite (IM7/977-3) in this work. The prediction is conducted for unnotched and open-hole specimens under tensile as well as compressive loading conditions. The coupons were categorized as unnotched tension (UNT), unnotched compression (UNC), open-hole tension (OHT), and open-hole compression (OHC). The investigated layups used in the DTDP program are [30, 60, 90, −60, −30]_2S_, [0, 45, 45, 90]_2S_, and [60, 0, −60]_3S_. 

A micromechanics-based FE model employing representative volume element (RVE) is developed to estimate the ultimate strength of composite laminates subjected to tensile and compressive loading. The mesoscale-based structural analysis was used to give input to the RVE regarding the mesoscale stresses. Damages that occurred in different composite constituents are observed based on the failure model for each constituent. The volume-based damage homogenization technique is used to avoid damage localization throughout the total matrix zone. The predicted results using MMF are also assessed by comparison with the experimental tests data as well as the results from the other analysis methods.

## 2. Micromechanics Based Progressive Damage Model

### 2.1. Multi-Scale Analysis

**Figure 1:**
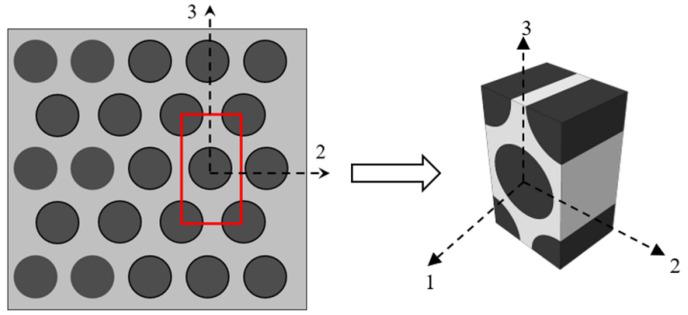
Representative volume element (hexagonal fiber).

The idealized model of the composite laminate is essential to predict their mechanics-based behavior subjected to static loading, prior to multi-scale analysis. The schematic of damage theory can be split up into macroscale procedure and microscale procedure. In the homogenized framework of multiscale analysis, the microscale sub-domain is a representative volume element (RVE) [29], from which micromechanical results are used to yield results from the macro-scale model. The RVE of a lamina is developed in the terms of the fiber, matrix, and interface to express microstructure as illustrated in Figure 1. 

The Representative Volume Element (RVE), a micro unit cell of hexagonal fiber shape as shown in Figure 1, may be utilized to correlate the behavior of ply to the behavior of fiber and matrix constituents [30]. Stress amplification factors (SAFs) [31], denoted by *M* and *A* matrices, are used for the transformation between the ply level macro stresses ($\bar{\sigma }$), and the fiber-based stresses (*σ**_f_***) and matrix-based stresses (*σ**_m_***) at the micro level. The transformation can be computed with the help of the following Equations (1) and (2).

(1) $$\sigma_{f}=M_{f}\bar{\sigma }+A_{f}\Delta T$$

(2) $$\sigma_{m}=M_{m}\bar{\sigma }+A_{m}\Delta T\;$$

where Δ*T* is the difference between ambient temperature and zero temperature. *M_f_* and *M_m_* are the SAFs (mechanical) for the fiber and matrix, respectively, whereas *A_f_* and *A_m_* are the SAFs (thermal) for the fiber and matrix, respectively. *M_f_* and *M_m_* are 6 × 6 matrices, and *A_f_* and *A_m_* are 6 × 1 matrices. Equation (1) shows that the correlation between stresses at macro level and micro level is linear with a uniform fiber distribution. 

Multiscale analysis is performed in three steps. First, the SAFs are computed by applying FE analysis on unit cell as shown in Figure 1. Second, Equation (1) is used to determine the micro level stresses of selected key points in the fiber and matrix using stresses in the ply. In the third step, micro stresses are then substituted in the constituent depended progressive damage model to determine the damage factors in the fiber and matrix. Later, as a result, the stiffness of each component, i.e., the fiber and matrix, was degraded, based on the calculated damages. Subsequently, the effective properties of the ply were again computed with the help of RVE.

### 2.2. Stress Amplification Factors

The diamond array of a fiber was selected to calculate the SAFs as shown in Figure 1. The stress state of unit cell is described by selecting 18 key points in the fiber and 19 key points in the matrix [28]. The majority of the selected points are positioned around the interface area where the maximum stress concentration takes place, and the remaining key points are situated in the center of the fiber and matrix. SAFs for the macro stress *M* and for the temperature increment *A* were calculated through direct FE analysis of a unit cell with appropriate repeated boundary conditions to ensure the correct behavior of a cell model [32]. The comprehensive structure of Equation (1) is as follows:

(3) $$\left(\begin{array}{c} \sigma_{1}\\ \sigma_{2}\\ \sigma_{3}\\ \sigma_{4}\\ \sigma_{5}\\ \sigma_{6} \end{array}\right)=\left[\begin{array}{cccccc} M_{11} & M_{12} & M_{13} & M_{14} & 0 & 0\\ M_{21} & M_{22} & M_{23} & M_{24} & 0 & 0\\ M_{31} & M_{32} & M_{33} & M_{34} & 0 & 0\\ M_{41} & M_{42} & M_{43} & M_{44} & 0 & 0\\ 0 & 0 & 0 & 0 & M_{55} & M_{56}\\ 0 & 0 & 0 & 0 & M_{65} & M_{66} \end{array}\right]\left(\begin{array}{c} {\bar{\sigma }}_{1}\\ {\bar{\sigma }}_{2}\\ {\bar{\sigma }}_{3}\\ {\bar{\sigma }}_{4}\\ {\bar{\sigma }}_{5}\\ {\bar{\sigma }}_{6} \end{array}\right)+\left(\begin{array}{c} A_{1}\\ A_{2}\\ A_{3}\\ A_{4}\\ A_{5}\\ A_{6} \end{array}\right)\Delta T$$

Stress amplification factors were determined by applying the uniformly distributed unidirectional unit loads one at a time at each direction to the unit cell shown in Figure 1.

For example, applying a unit load having uniform distribution at x direction of unit cell with no thermal load will result in the simplification of Equation (3). The simplified Equation (4) is given below:

(4) $$\left(\begin{array}{c} \sigma_{1}\\ \sigma_{2}\\ \sigma_{3}\\ \sigma_{4}\\ \sigma_{5}\\ \sigma_{6} \end{array}\right)=\left[\begin{array}{cccccc} M_{11} & M_{12} & M_{13} & M_{14} & 0 & 0\\ M_{21} & M_{22} & M_{23} & M_{24} & 0 & 0\\ M_{31} & M_{32} & M_{33} & M_{34} & 0 & 0\\ M_{41} & M_{42} & M_{43} & M_{44} & 0 & 0\\ 0 & 0 & 0 & 0 & M_{55} & M_{56}\\ 0 & 0 & 0 & 0 & M_{65} & M_{66} \end{array}\right]\left(\begin{array}{c} 1\\ 0\\ 0\\ 0\\ 0\\ 0 \end{array}\right)$$

The solution of linear system given in Equation (4) yields Equation (5):

(5) $$\left(\begin{array}{c} \sigma_{1}\\ \sigma_{2}\\ \sigma_{3}\\ \sigma_{4}\\ \sigma_{5}\\ \sigma_{6} \end{array}\right)=\left[\begin{array}{c} M_{11}\\ M_{21}\\ M_{31}\\ M_{41}\\ 0\\ 0 \end{array}\right]$$

Equation (5) shows that the calculated SAFs are the micromechanical stresses, e.g., *M*_11_ is basically the $\sigma_{1}$ and so on. The same method was applied to other directions to calculate all the other SAFs. 

### 2.3. Damage Model of Constituents

A separate damage model is used for each individual constituent of the composite material, i.e., fiber and matrix, due to their different mechanical properties and behaviors. Usually, the glass fiber behaves as an isotropic material, and carbon fiber behaves as a transversely isotropic. Therefore, the behavior of both carbon and glass fibers are presumed to be linear elastic and brittle. On the other hand, the polymetric matrix behaves as an isotropic and ductile material, demonstrating non-linear behavior when it is damaged.

### 2.4. Fiber Failure Criteria

For fiber material, the maximum longitudinal stress damage model was used as (Equation (6)):

(6) $$-C_{f}<\sigma_{f11}<T_{f}$$

**Figure 2:**
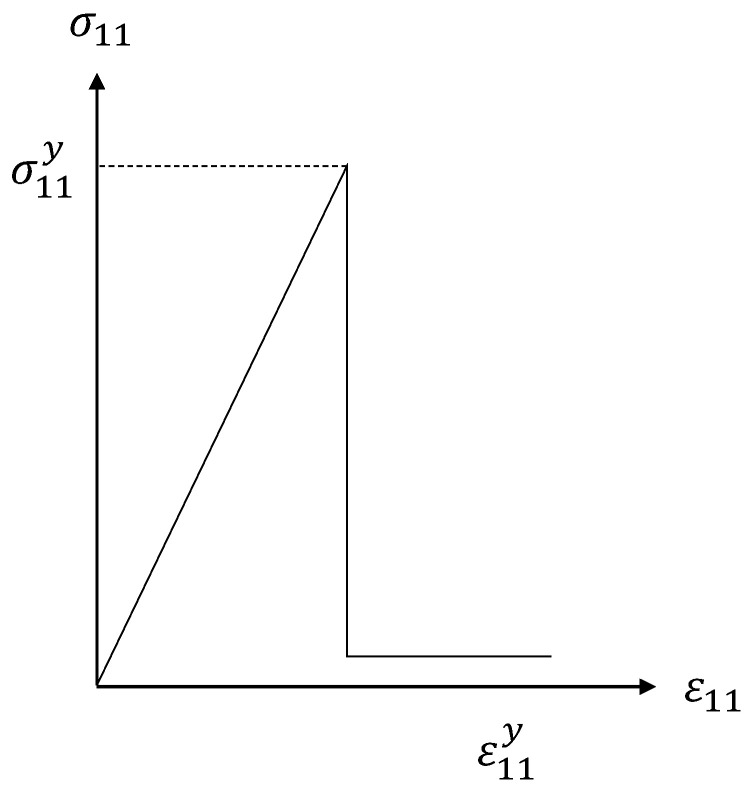
Material damage model for fiber.

The symbol $\sigma_{f11}$ indicates the longitudinal stress at the micro level for the fiber, and notations *T_f_* and *C_f_* presents the tensile strength (longitudinal) and compressive strength (longitudinal) of the fiber constituent, respectively. As soon as the produced stress in the longitudinal direction of fiber element reaches the *T_f_* or *C_f_*, then the fiber will fail. As a result of failure, the stiffness of fiber constituent will be significantly degraded as showed in the progressive model Figure 2. 

Therefore, once the fiber failure occurs, the localized damage factor, *D_f_*, is set to 0.9 to ensure computational stability [33], and if fiber failure does not occur, then the *D_f_* is assigned value of zero. The fiber materials in this study are taken as brittle; therefore, complete damage of single element is considered catastrophic towards the total area of the fiber. As a result, the global factor for damage in the fiber was described as the maximum *D_f_* of all the calculated *D_f_* of elements (Equation (7)).

(7) $${\bar{D}}_{f}=Maximum\;\left(D_{f}^{\left(j\right)}\right)$$

where $D_{f}^{\left(j\right)}$ is the damage factor produced in jth element of fiber. Therefore, a fundamental correlation for the stresses induced in the fiber was then calculated as (Equation (8)):

(8) $$\sigma_{f}=\left(1-{\bar{D}}_{f}\right)\;C_{f}\varepsilon_{f}$$

In Equation (8), *C_f_* indicates the stiffness matrix of the total fiber area inside the RVE.

### 2.5. Matrix Failure Criteria

In the majority of cases, the matrix materials are isotropic, with different tensile and compressive strengths. Both types of strength contribute in a different way to matrix failure. Raghava et al. [34] and Ha et al. [26] presented a tailored adaptation of the von-Mises criterion for failure, using tensile strength *T_m_* and compressive strength *C_m_*, which is as follows (Equation (9)):

(9) $$\frac{\sigma_{VM}^{2}}{C_{m}T_{m}}+\left(\frac{1}{T_{m}}-\frac{1}{C_{m}}\right)I_{1}=1$$

*I*_1_ and *σ_VM_* may be computed by utilizing stress-based components with the help of the subsequent Equations (10)–(12):

(10) $$I_{1}=\sigma_{m1}+\sigma_{m2}+\sigma_{m3}$$

(11) $$\sigma_{VM}=\sqrt{\frac{1}{2}\left[\left[{\left(\sigma_{m1}-\sigma_{m2}\right)}^{2}\right]+\left[{\left(\sigma_{m2}-\sigma_{m3}\right)}^{2}\right]+\left[{\left(\sigma_{m3}-\sigma_{m1}\right)}^{2}\right]+\sigma_{m4}^{2}+\sigma_{m5}^{2}+\sigma_{m6}^{2}\right]}$$

The failure criterion mentioned in Equation (9) is the same as the condition that an equivalent stress $\sigma_{eq}$ achieves the initial *T_m_*,

(12) $$\sigma_{eq}=\frac{\left(\beta -1\right)\;I_{1}+\sqrt{\left(\beta -1\right){\;}^{2}\;I_{1}^{2}+4\beta \sigma_{VM}^{2}}}{2\beta }$$

In Equation (12), β for the matrix is defined as the ratio between compressive strength and tensile strength. The equivalent stress $\sigma_{eq}$_,_ frequently termed stasis stress, is established upon the stress–strain correlation. The $\sigma_{eq}$ can be translated into the equivalent strain $\varepsilon_{eq}$ with the help of subsequent Equation (13).

(13) $$\varepsilon_{eq}=\frac{\left(\beta -1\right)J_{1}+\sqrt{{\left(\beta -1\right)}^{2}J_{1}^{2}+{\left(\frac{2-4\nu }{1+\nu }\right)}^{2}\beta \varepsilon_{VM}^{2}}}{2\beta \left(1-2\nu \right)}$$

In Equation (13), J_1_ represents first-strain invariant, $\varepsilon_{VM}$ represents the von Mises equivalent strain and *ν* is the Poisson’s ratio. *J_1_* and $\varepsilon_{VM}$ can be computed with the help of strain-based components using the subsequent Equations (14) and (15) [35]:

(14) $$J_{1}=\varepsilon_{m1}+\varepsilon_{m2}+\varepsilon_{m3}$$

(15) $$\varepsilon_{VM}=\sqrt{\frac{1}{2}\left[\left[{\left(\varepsilon_{m1}-\varepsilon_{m2}\right)}^{2}\right]+\left[{\left(\varepsilon_{m2}-\varepsilon_{m3}\right)}^{2}\right]+\left[{\left(\varepsilon_{m3}-\varepsilon_{m1}\right)}^{2}\right]+\varepsilon_{m4}^{2}+\varepsilon_{m5}^{2}+\varepsilon_{m6}^{2}\right]}$$

**Figure 3:**
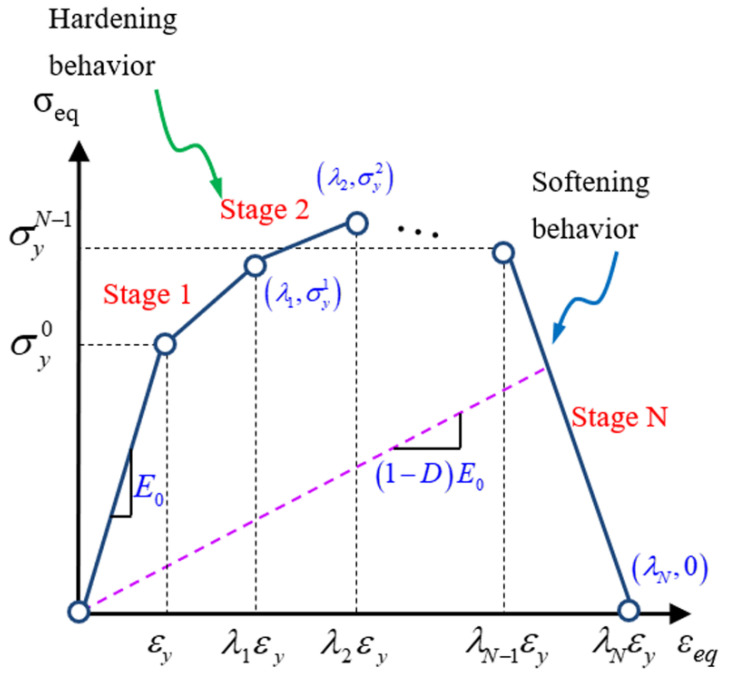
Material damage model for the matrix [27].

The damage evolution in the matrix material was computed with the help of the equivalent strain calculated from Equation (13). Using $\sigma_{eq}$ and $\varepsilon_{eq}$, a multilinear failure model for a matrix, based on stress strain, was suggested by Ha et al. [28], as shown in Figure 3. The response of the matrix, before damage, follows the linear stress–strain relation. As soon as the damage occurs, the matrix reveals hardening behavior, subsequently followed by softening behavior. The stiffness degradation occurs on the basis of the damage factor *D_m_* in both hardening and softening behavior. The overall damage factor in the matrix *D_m_* can be computed using the following Equation (16).

(16) $$\begin{array}{c} D_{m}=1-\frac{\left(\varepsilon_{y}^{\left(i\right)}-\varepsilon_{eq}\right)\sigma_{y}^{\left(i-1\right)}+\left(\varepsilon_{eq}-\varepsilon_{y}^{\left(i-1\right)}\right)\sigma_{y}^{\left(i\right)}}{E_{0}\left(\varepsilon_{eq}-\varepsilon_{y}^{\left(i-1\right)}\right)\varepsilon_{eq}}\\ \left(\varepsilon_{y}^{\left(i-1\right)}<\varepsilon_{eq}<\varepsilon_{y}^{\left(i\right)}\right) \end{array}$$

where $\sigma_{y}^{\left(i-1\right)}$ and $\varepsilon_{y}^{\left(i-1\right)}$ indicate the yielding stress and strain at the initial point of the ith damage state; $\sigma_{y}^{\left(i\right)}$ and $\varepsilon_{y}^{\left(i\right)}$ indicate the yielding stress and strain at the ending point of the ith damage state; $\varepsilon_{eq}$ represents existing equivalent strain in the *i*-th damage state, and *E*_0_ indicates undamaged matrix’s stiffness. To calculate the yielding stress and strain at the start and end point of the damage state, the tensile stress–strain curve of the polymer matrix (i.e., epoxy 977-3) reported in the literature [36] is used in this work. 

The stress–strain curve is divided into 10 stages. In each stage, the yield stress and corresponding yield strain are used to evaluate the matrix damage *D_m_*. The details regarding the yield stress–strain data of each stage are described in Section 3.3. The maximum *D_m_* was selected as 0.9 to ensure computational stability [33]. After the computation of each element’s failure state, the damage localization was prevented with the help of the volume-based damage homogenization technique [37], given by the following Equation (17).

(17) $${\bar{D}}_{m}=\frac{\int_{V}{\left[D_{m}\right]}^{p}\;d\;V}{\int_{V}\;d\;V}$$

In Equation (17), *p* is a local damage’s positive weighting parameter and *V* is defined as the total volume of the matrix area inside the RVE. Considering the stiffness degradation of the matrix, the constitutive relation was calculated by (Equation (18)):

(18) $$\sigma_{m}=\left(1-{\bar{D}}_{m}\right)\;C_{m}\varepsilon_{m}$$

In Equation (18), *C_m_* indicates the stiffness matrix of the total matrix area in the RVE.

### 2.6. Numerical Implementation of Constituent Damage Models

**Figure 4:**
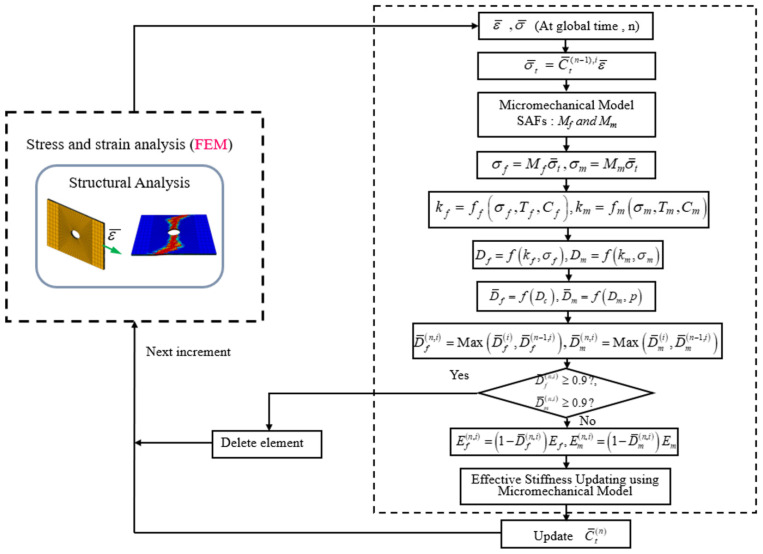
Algorithm combining the MMF and the progressive damage model.

Figure 4 shows the flow diagram of the algorithm, established for the methodology combining MMF and progressive damage model. The detailed methodology is as follows:

1. The overall global strain produced at the global time *n*, was calculated through the addition of the global strain increment at *n* and at the previous step *n-1*.
2. With regard to the composite laminate, the macro level stresses of each constituent element, i.e., the fiber and matrix, were calculated with the help of previous effective stiffness properties.
3. Subsequently, the micro level stress of each constituent was computed from the macro stresses with the help of SAFs.
4. The damage model of constituents was then employed to both fiber and matrix to calculate the damage factor in the fiber and matrix areas, symbolized by *D_m_* and *D_f_*.
5. The total damage factor produced for matrix and fiber zones were then analyzed using their corresponding damage methods, i.e., the maximum damage for fiber and the damage homogenization using volume-based technique for the matrix.
6. The stiffness degradation of matrix and fiber were calculated using the status of the total damage factor. Subsequently, the ply level effective properties were calculated for the following time increment.
7. The numerical execution of MMF was implemented using ABAQUS, combined with the user subroutine USDFLD.

## 3. Methodology

An implicit scheme was employed to execute the simulation of the static loading with the help of the finite element analysis software package ABAQUS/Implicit.

### 3.1. Models Geometries and Boundary Conditions

**Figure 5:**
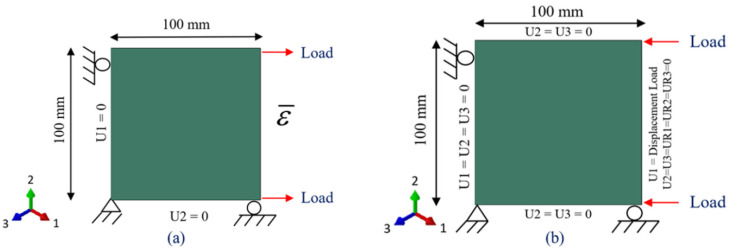
Geometries and boundary conditions (**a**) UNT (**b**) UNC.

**Figure 6:**
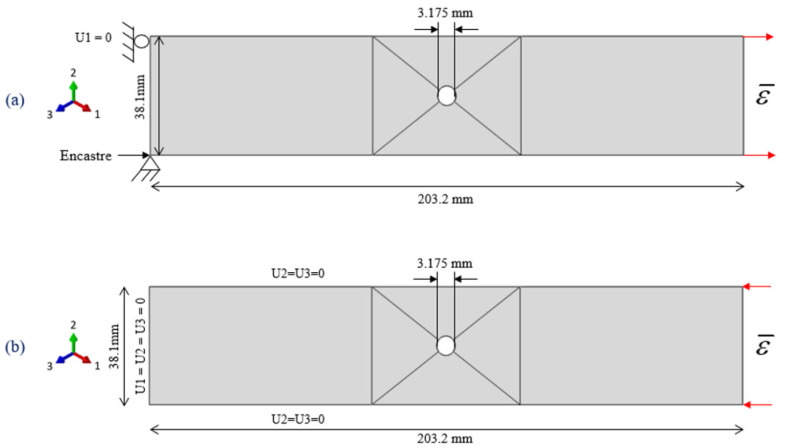
Geometries and boundary conditions (**a**) OHT (**b**) OHC.

The DTDP exercise program by AFRL included UNT, UNC, OHT, and OHC, and there were three layups for each coupon, resulting in twelve multi-directional configurations. The investigated layups were soft layup [30, 60, 90, −60, −30]_2S_, quasi-isotropic layup [0, 45, 90, −45]_2S_, and [60, 0, −60]_3S_ with 0° developed in parallel with the loading direction. The model geometries along with dimensions and boundary conditions for UNT and UNC is given Figure 5 and for OHT and OHC is given in Figure 6. 

To avoid unnecessary complexity to the finite element models and artificial stress concentrations, the geometrical models were simplified by exclusion of the fixtures. In the case of UNT ad UNC, only the gage section was modeled, and boundary conditions were applied to the gage section as shown in Figure 5. For all developed models, the ply thickness was taken as 0.12954 mm.

### 3.2. Mesh Details

The successful simulation of physical damage mechanisms occurring in composite structures as a result of finite element-based failure theory is highly reliant on the consistency of the mesh discretization [38]. Modeling and meshing laminate using single element in the direction of stack, demonstrating entire composite laminate, will no longer be able to simulate the state of interlaminar damage. Ideally, the finite element model with structured mesh type will have complete reliability to catch and identify all the failure modes; however, using this type of mesh will result in the overcapacity of computing resources as well as consumption of excessive time to complete the simulation with full load history. Therefore, a workable approach to create a finite element model is one that produces precise simulations without being excessively cumbersome.

In this study, solid element type is used with through-thickness discretization techniques, which utilize non-layered elements. In structured mesh, non-layered types of elements have a single ply per element and are given preference when models have stress concentrations causing large transverse shear and normal stress gradients. Non-layer types of elements are also capable of simulating the localized softening behavior that arises because of failure occurs in the fiber and matrix [39]. As half of the specimens investigated in the DTDP exercise by AFRL contains stress concentrations due to notches, the meshes of all the models used non-layered elements, i.e., each ply was meshed with the help of separate layer.

**Figure 7:**
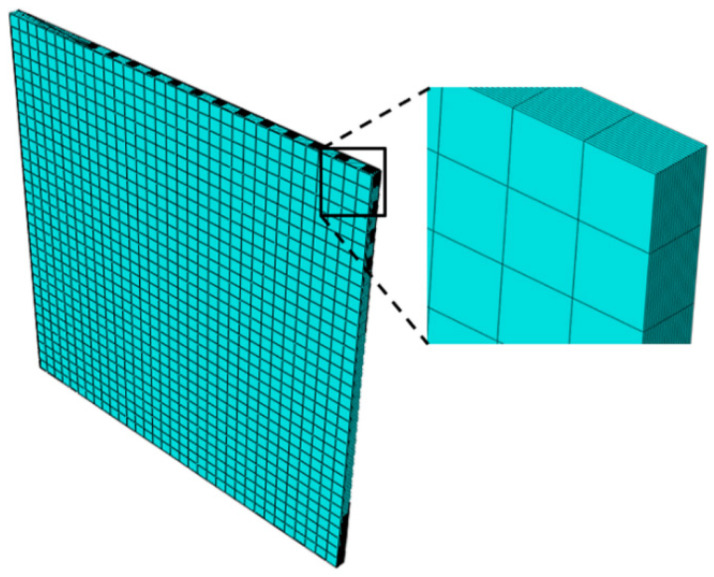
Mesh adapted for UNT and UNC models.

In finite element models, the plies were modelled using the Abaqus element type C3D8R, which is an eight-node linear brick, reduced integration, hourglass control element [40]. The reduced integration elements are efficient, specifically in the case of progressive damage simulations as the entire element is represented with the help of a single integration point [41]. The adapted mesh developed for the unnotched models is illustrated in Figure 6. The mesh discretization for the notched models as illustrated in the Figure 7, is based on “notch centric” design, characterized with the help of rounded mesh all around the region of notch that rapidly converts into a rectangular-based mesh shifting farther from the notch. 

**Figure 8:**
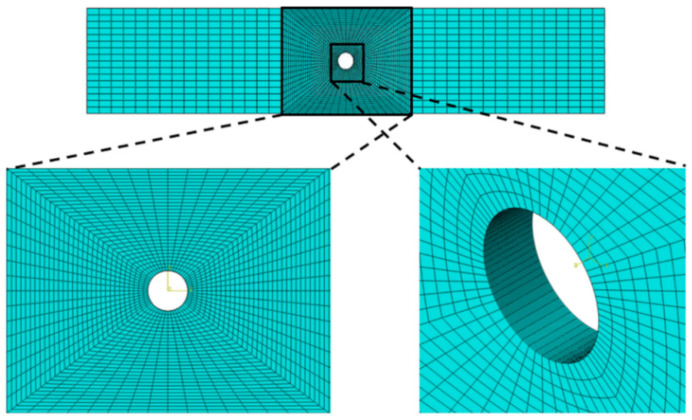
Mesh adapted for OHT and OHC models.

A zoomed view of the mesh is also shown in Figure 7 and Figure 8. Mesh sensitivity analysis was performed for all the cases, and we established that the final adapted mesh was to be a compromise between the output quality and time required for computation for each case. In particular, for the mesh sensitive OHT and OHC coupons, five cases were investigated with the numbers of elements of 40, 960; 67, 840; 97, 280; 179, 200; and 430, 080 elements, respectively. The mesh convergence study shows that there is no major difference in the strength values as the number of elements are increased; therefore, the case with number of elements of 97, 280, was chosen for the study, keeping in mind the time required and the output quality.

### 3.3. Material Properties

In this study, the matrix (977-3) is presumed as an isotropic material with two independent material properties, and the fiber (IM7) behaves as transversely isotropic with five independent material properties. Initially, the mechanical properties of IM7/977-3 ply were taken from experimental results provided by the AFRL [14]. The lamina experimental tests were all performed employing ASTM test standards 85. During the benchmark study, the initial measurement of the compressive failure strength of laminate that was delivered to the analysts did not keep into account the specimen buckling, resulting in an incorrectly measured failure load. However, after the discovery of buckling failure mode, analysts were instructed to increase the laminate compressive failure strength input property to 1680 MPa for recalibration [14].

The calibration of material properties for the carbon fiber (IM7) and matrix (977-3) was performed using the micromechanics approach, which minimizes the difference between the numerical model and experimental data. During calibration, the fiber and matrix properties were determined using the micromechanics model to estimate the composite laminate elastic properties that closely matched the experimental results. The stiffness and strength properties of the carbon-based fiber constituent [14] and matrix listed in Table 1. The tensile yield stress and yield strain data of matrix (977-3) [36] at different stages for matrix multi-linear damage model is given in Table 2. The effective properties of the ply were computed from the micro unit cell, using the micromechanics approach and are listed in Table 3.

## 4. Results and Discussion

The ability to precisely predict the damage onset and damage propagation have a potential role in designing and manufacturing composite structures. Furthermore, accurate progressive damage models could substantially minimize the constituents damage tolerance tests, reducing the time and cost to verify a composite structure. This section investigates the performance of the proposed micromechanics-based failure model (MMF) with experimental results [14] and other failure models [21]. First, the finite element simulations were performed for all four test specimens, i.e., UNT, UNC, OHT, and OHC, with three distinct multi-directional layups ([30, 60, 90, −60, −30]_2S_, [0, 45, 90, −45]_2S_, and [60, 0, −60]_3S_) per coupon. The findings were then compared with the experimental results and other failure techniques to investigate the efficiency of the MMF.

### 4.1. Unnotched Tension

**Figure 9:**
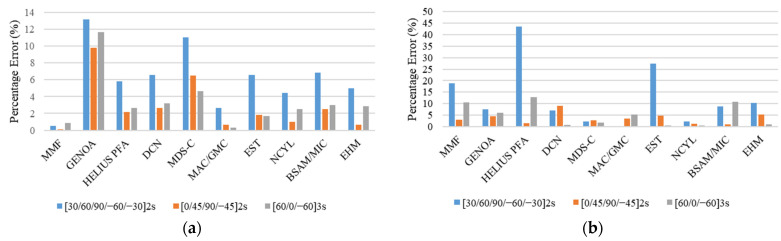
Comparison of simulation with experimental results for (**a**) unnotched tension stiffness and (**b**) unnotched tension static strength.

The comparison between the experimental and simulated results for static strength at failure and stiffness for different unnotched tension specimen/layup configurations is given in Table 4. The comparison of results of MMF and other analysis methods [21] with experimental results [14] for unnotched static strength and stiffness in terms of percentage error is given in the form of bar charts in Figure 9.

The results given in Table 4 and Figure 9 show that the MMF presented a good agreement for quasi-isotropic [0, 45, 90, −45]_2S_ configuration and [60/0/−60]_3S_ configuration with a percentage error of 3.12% and 10.45%, respectively. Whereas, for soft layup [30/60/90/−60/−30]_2S_, the MMF underpredicted the results with a percentage error of 18.82 %. The higher percentage error in the simulation response of this soft layup is attributed to the fact that the real failure mechanism for the soft layup as per experimental investigation is too complicated to detect using modeling techniques. 

**Figure 10:**
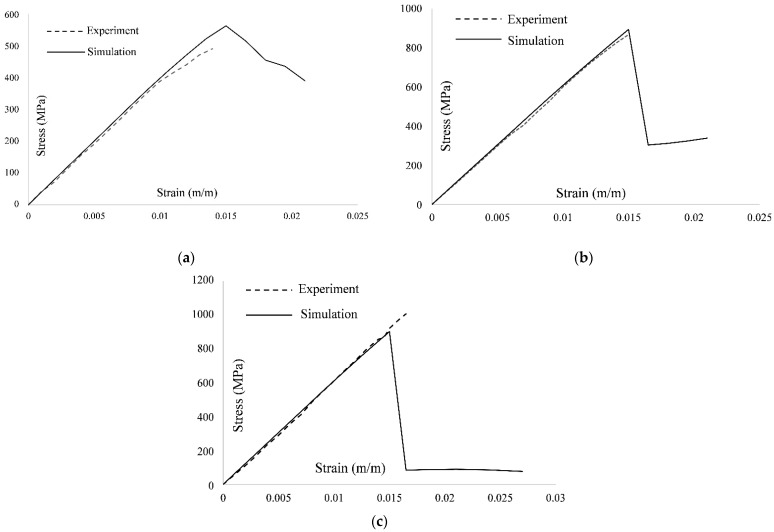
Comparison of the experimental and simulation responses for UNT static strength of (**a**) [30/60/90/−60/−30]_2S_, (**b**) [0/45/90/−45]_2S_, (**c**) [60/0/−60]_3S_ coupons.

This issue was also discussed by Dalgarno et al. [17]. The investigation of experimental results and failure images [14] indicate that the laminate exhibited considerable delamination. Currently, MMF does not account for the delamination effect due to computational simplicity and execution speed. To clearly understand the overall progressive damage simulation response, Figure 10 shows a comparison of the stress–strain curves obtained by MMF and the experimental results for the UNT case for all three layups.

In addition to the unnotched tension strength, the comparison of the unnotched tension stiffness results with experimental results given in Table 4 and Figure 9 shows that the MMF exhibited excellent agreement for [30/60/90/−60/−30]_2S_, [0, 45, 90, −45]_2S_, and [60/0/−60]_3S_ layups with percentage errors of 0.53%, 0.08%, and 0.83%, respectively.

The comparison of stiffness results in Figure 9 shows that the MMF performed better than the other failure methods. However, for the strength prediction, soft layup [30, 60, 90, −60, −30]_2S_, MMF, and other analysis methods, like HELIUS PFA, EST, BSAM/MIC, and EHM, have higher percentage errors. For the other two layups, i.e., [0, 45, 90, −45]_2S_ and [60, 0, −60]_3S_, MMF performed reasonably well to predict the unnotched static strength.

### 4.2. Unnotched Compression

**Figure 11:**
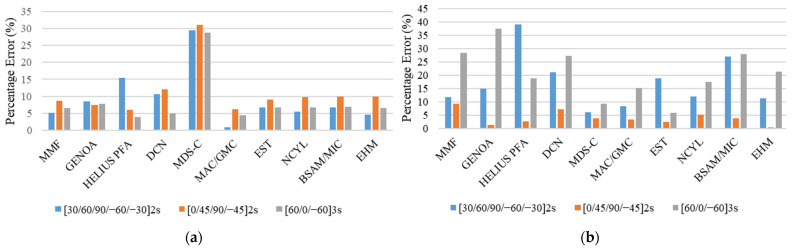
Comparison of simulation with experimental results for (**a**) unnotched compression stiffness and (**b**) unnotched compression static strength.

The results of the unnotched compression static strength and stiffness are given in Table 5. A comparison was performed between the experimental and simulated results for static strength and stiffness for three different layups, which were [30, 60, 90, −60, −30]_2S_, [0, 45, 90, −45]_2S_ and [60, 0, −60]_3S_. For the UNC case, the comparison of MMF and other analysis methods with experimental results for the three investigated layups is given in Figure 11.

**Figure 12:**
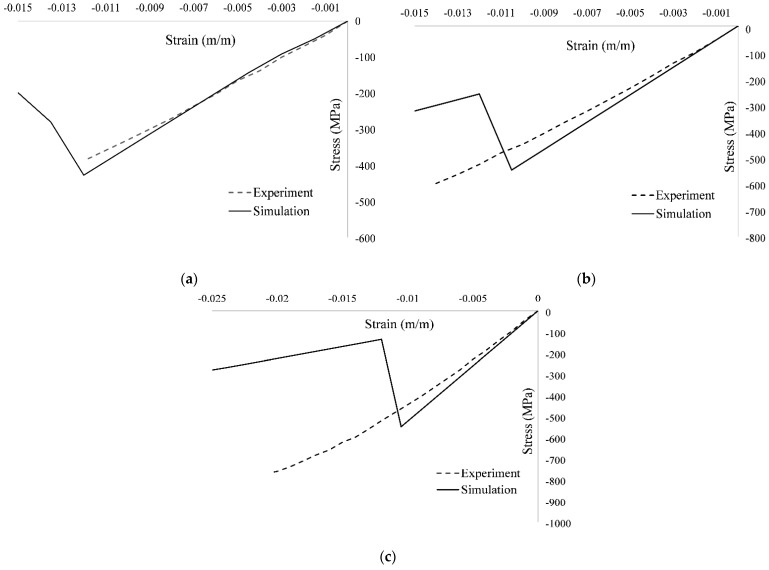
Comparison of the experimental and simulation responses for UNC static strength of (**a**) [30/60/90/−60/−30]_2S_, (**b**) [0/45/90/−45]_2S_, (**c**) [60/0/−60]_3S_ coupons.

Examination of the values of static strength at failure for different configurations given in Table 5, and Figure 11 reveals a good agreement between MMF and the experimental results for [30, 60, 90, −60, −30]_2S_ and [0, 45, 90, −45]_2S_ with percentage errors of 11.78% and 9.29%, respectively. However, for [60, 0, −60]_3S_, the results of MMF are underpredicted with a percentage error of 28.37%. In addition, the comparison of simulated response by MMF and experimental response in terms of the stress–strain curves for the UNC case for all three layups is shown in Figure 12.

On the other hand, for unnotched compression stiffness, the results of MMF are well aligned with the experimental results with the percentage errors of 5.15%, 8.71%, and 6.61% for the [30, 60, 90, −60, −30]_2S_, [0, 45, 90, −45]_2S_, and [60, 0, −60]_3S_ layups, respectively.

For the stiffness comparison shown in Figure 11, the performance of MMF and other analysis methods is excellent except for the MDS-C, in which the percentage error is very high for all investigated layups. For the strength prediction in the UNC case, the comparison showed a good performance of MMF for the [30, 60, 90, −60, 30]_2S_ and [0, 45, 90, −45]_2S_ layups. However, for the [60, 0, −60]_3S_ laminate sequence, most of the analysis methods, including MMF, have higher percentage errors as shown in Figure 11.

### 4.3. Notched Tension

**Figure 13:**
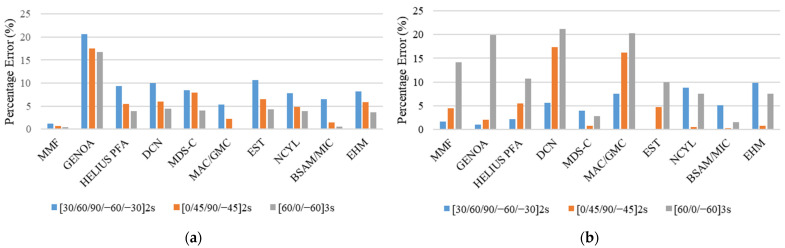
Comparison of simulation with experimental results for (**a**) open hole tension stiffness and (**b**) open hole tension static strength.

The experimental and simulated results for the notched pattern coupons are given in Table 6. The results show the comparison between the experimental and simulated results for three investigated multidirectional composite layups. The comparison of MMF and other failure criterion with MMF for open hole tension static strength at failure and stiffness is illustrated in the form of bar charts in Figure 13.

**Figure 14:**
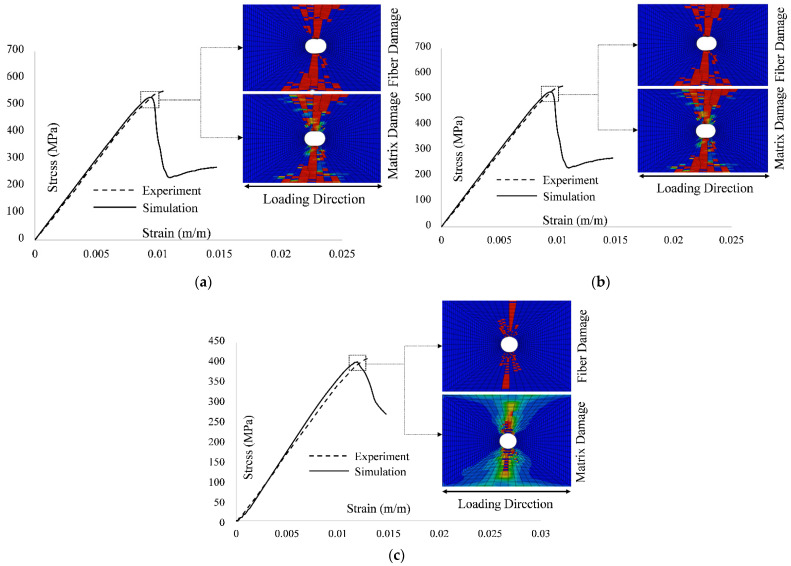
Comparison of the experimental and simulation responses along with images of simulated fiber and matrix damage distribution at failure for OHT static strength of (**a**) [30/60/90/−60/−30]_2S_, (**b**) [0/45/90/−45]_2S_, (**c**) [60/0/−60]_3S_ coupons.

From the results presented in Table 6 and Figure 13, it is concluded that the MMF model correlated better to experimental findings for the open hole tension static strength for the soft layup [30, 60, 90, 60, −30]_2S_ and quasi-isotropic lay [0, 45, 90,−45]_2S_ with a percentage error of 1.71% and 4.51%, respectively. However, for the [60, 0, −60]_3S_, the results of static strength at failure are underpredicted with a percentage error of 14.18%. To clearly recognize the overall progressive damage simulation response and accumulated simulated damage at failure, Figure 14 shows a comparison of stress–strain curves obtained by MMF and experimental results for the OHT case for all three layups along with the images of simulated fiber and matrix damage distribution at failure.

On the other hand, the results of MMF for notched tension stiffness are well aligned with the experimental results for [30, 60, 90, −60, −30]_2S_, [0, 45, 90, −45]_2S_, and [60, 0, −60]_3S_ layups with percentage errors of 1.25%, 0.62%, and 0.41%, respectively.

Like the previous two cases of UNT and UNC, MMF performed well compared to the other analysis methods for OHT stiffness. For the strength prediction case, the MMF showed good agreement for the [30, 60, 90, −60, −30]_2S_ and [0, 45, 90, −45]_2S_ layups. However, for the [60, 0, −60]_3S_ layup, like the UNC case, nine out of ten analysis methods, including MMF, showed high percentage errors, as shown in Figure 13.

### 4.4. Notched Compression

**Figure 15:**
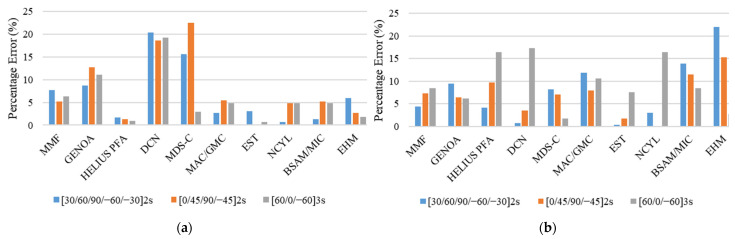
Comparison of simulation with experimental results for (**a**) open hole compression stiffness and (**b**) open hole compression static strength.

Table 7 shows a comparison of overall open hole compression static strength and stiffness responses of simulation with the experimental results for the specimen of soft layup [30, 60, 90, −60, −30]_2S_, quasi-isotropic layup [0, 45, 90, −45]_2S_, and [60, 0, −60]_3S_ layup. For the OHC case, Figure 15 compares percentage errors for the MMF and other analysis methods with experimental results for the three investigated composite layups.

**Figure 16:**
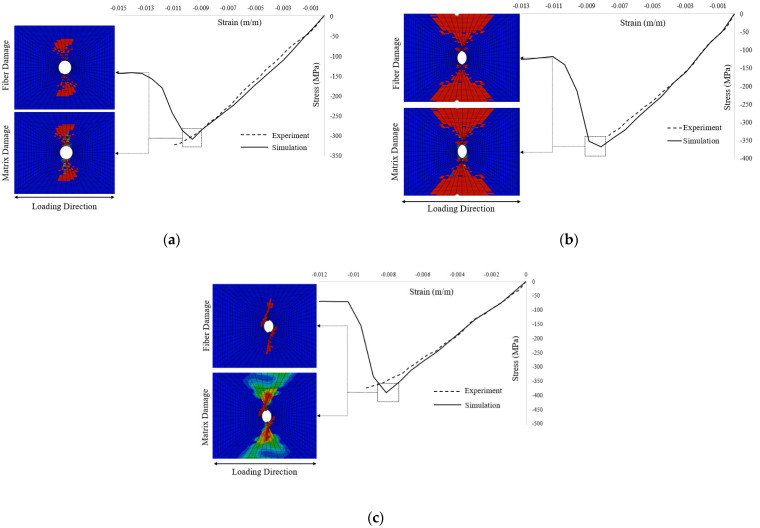
Comparison of the experimental and simulation responses along with images of simulated fiber and matrix damage distribution at failure for OHC static strength of (**a**) [30/60/90/−60/−30]_2S_, (**b**) [0/45/90/−45]_2S_, (**c**) [60/0/−60]_3S_ coupons.

The results given in Table 7 and Figure 15 show that the MMF exhibited good agreement with the obtained experimental results for all the experimentally investigated composite layups. For open hole compression static strength, the percentage errors for [30, 60, 90, 60, −30]_2S_, [0, 45, 90, −45]_2S_, and [60, 0, −60]_3S_ layups are 4.41%, 7.33%, and 8.38%, respectively. On the other hand, for the open hole compression stiffness, the percentages for the layups are 7.67%, 5.23%, and 6.36%, respectively. In addition, the comparison of the stress–strain curves obtained by MMF and the experimental results for the OHC case for all three layups along with the images of simulated fiber and matrix damage distribution at failure is shown in Figure 16.

The comparison given in Figure 15 showed that the MMF performed reasonably well to predict the notched compression static strength and stiffness compared to most of the other analysis methods. For OHC stiffness prediction, the GENOA, DCN, and MDS-C analysis methods had high average percentage errors. For the OHC strength prediction, there was no clear pattern for the prediction of the result, as shown in Figure 15. However, MMF performed well for the prediction of strength for all three investigated layups.

## 5. Conclusions

In this study, a micromechanics-based progressive damage model MMF was used to predict the ultimate strength at failure and stiffness of four different multi-directional laminated composite test specimens, i.e., UNT, UNC, OHT, and OHC. For each case, three different composite laminate layups were used: [30, 60, 90, −60, −30]_2S_, [0, 45, 90, −45]_2S_, and [60, 0, −60]_3S_. The results of MMF were then compared with the experimental results provided by AFRL and the results of nine different analysis methods that participated in the DTDP program. 

The simulations were performed using ABAQUS, and the numerical implementation of MMF was done using the user subroutine USDFLD. The simulated results of stiffness for all 12 specimens/layups accurately aligned with the experimental results. For the ultimate strength at failure predictions, the simulated results agreed extremely well with the experimental results for the quasi-isotropic [0, 45, 90, −45]_2S_ layup with the percentage errors within 10% of the measured failure loads. 

For the [60, 0, −60]_3S_ configuration, the findings of MMF were underpredicted with percentage errors of 28.37% and 14.18% for the UNC and OHT, respectively. For the soft layup [30, 60, 90, −60, −30]_2S_, the simulated results underpredicted the strength of the UNT specimen by 18.82%. For this specific laminate, the experimentally detected failure pattern observed substantial delamination during investigation. The proposed modeling technique does not take into consideration the delamination, which resulted in the higher percentage error. We concluded that MMF demonstrated the ability to correctly predict the ultimate strength at failure for a range of laminates under tensile and compressive loading.

## Figures and Tables

**Table 1:** Material properties of the carbon fiber (IM7) and matrix (977-3).

Material Properties	Value
**Carbon Fiber (IM7)**	
Longitudinal Tensile Modulus *E*_*f*1_ (GPa)	276
Transverse Tensile Modulus *E*_*f*2_ (GPa)	21
Longitudinal Compressive Modulus *E*_*f*1_ (GPa)	204
Transverse Compressive Modulus *E*_*f*2_ (GPa)	21
In-plane Shear Modulus *G*_*f*12_ (GPa)	29
Transverse Modulus *G*_*f*23_ (GPa)	7
Poisson’s ratio *v*_*f*12_	0.31
Poisson’s ratio *v*_*f*23_	0.28
Longitudinal Tensile strength *X_F_* (MPa)	4082
Longitudinal Compressive strength *X_FP_* (MPa)	2682
Fiber volume fraction	0.592
**Matrix (977-3)**	
Elastic modulus *E_m_* (GPa)	3.5
Elastic Poisson’s ratio *v_m_*	0.35
Tensile strength *X_M_* (MPa)	81
Compressive strength *X_MP_* (MPa)	131

**Table 2:** Tensile yield stress and yield strain data of the matrix (977-3) at different stages for the multi-linear damage model for the matrix.

Stages	Yield Stress (MPa)	Yield Strain (m/m)
Stage 1	82.16	0.03
Stage 2	100.83	0.08
Stage 3	113.15	0.16
Stage 4	119.50	0.30
Stage 5	124.73	0.45
Stage 6	131.45	0.62
Stage 7	135.93	0.76
Stage 8	142.66	0.92
Stage 9	146.76	1.06
Stage 10	153.86	1.21

**Table 3:** Material properties of laminae (IM7/977-3).

Material Properties	Value
Longitudinal Tensile Modulus *E*_*f*1_ (GPa)	164
Transverse Tensile Modulus *E*_*f*2_ (GPa)	8.9438
Longitudinal Compressive Modulus *E*_*f*1_ (GPa)	121.6
Transverse modulus *E*_*f*2_ (GPa)	8.9236
In-plane Shear Modulus *G*_*f*12_ (GPa)	4.3516
Transverse Modulus *G*_*f*23_ (GPa)	2.9906
Poisson’s ratio *v*_*f*12_	0.324
Poisson’s ratio *v*_*f*23_	0.417

**Table 4:** Unnotched tension results.

		Unnotched Tension Static Strength			Unnotched Tension Stiffness			
Team	Static Strength	[30, 60, 90, −60, −30]_2S_	[0, 45, 90, –45]_2S_	[60, 0, −60]_3S_	Elastic Modulus	[30, 60, 90, −60, −30]_2S_	[0, 45, 90, −45]_2S_	[60, 0, −60]_3S_
Experiment	σ_max_ (Mpa)	473	866	1005	E (Gpa)	38	60	60
MMF	σ_max_ (Mpa)	562	893	900	E (Gpa)	37.8	59.95	60.5
GENOA	σ_max_ (Mpa)	508	828	944	E (Gpa)	43.0	65.9	67.0
Helius PFA	σ_max_ (Mpa)	679	879	877	E (Gpa)	40.2	61.3	61.6
DCN	σ_max_ (Mpa)	506	944	997	E (Gpa)	40.5	61.6	61.9
MDS-C	σ_max_ (Mpa)	462	890	987	E (Gpa)	42.2	56.1	62.8
MAC/GMC	σ_max_ (Mpa)	474	897	951	E (Gpa)	39.0	59.6	59.8
EST	σ_max_ (Mpa)	603	825	1009	E (Gpa)	40.5	61.1	61.0
NCYL	σ_max_ (MPa)	462	856	1011	E (GPa)	39.7	60.6	61.5
BSAM/MIC	σ_max_ (MPa)	432	858	1113	E (GPa)	40.6	61.5	61.8
EHM	σ_max_ (MPa)	522	911	1014	E (GPa)	39.9	60.4	61.7

**Table 5:** Unnotched compression results.

		Unnotched Compression Static Strength			Unnotched Compression Stiffness			
Team	Static Strength	[30, 60, 90, −60, −30]_2S_	[0, 45, 90, −45]_2S_	[60, 0, −60]_3S_	Elastic Modulus	[30, 60, 90, −60, −30]_2S_	[0, 45, 90, −45]_2S_	[60, 0, −60]_3S_
Experiment	*σ_max_* (MPa)	382	603	765	E (GPa)	33	48	49
MMF	*σ_max_* (MPa)	427	547	548	E (GPa)	34.7	52.18	52.24
GENOA	*σ_max_* (MPa)	439	610	479	E (GPa)	30.2	44.4	45.2
Helius PFA	*σ_max_* (MPa)	531	619	622	E (GPa)	38.1	50.9	50.9
DCN	*σ_max_* (MPa)	462	560	557	E (GPa)	36.5	53.8	51.4
MDS-C	*σ_max_* (MPa)	405	625	836	E (GPa)	42.7	62.9	63.1
MAC/GMC	*σ_max_* (MPa)	350	583	649	E (GPa)	33.3	51	51.2
EST	*σ_max_* (MPa)	454	618	720	E (GPa)	35.2	52.3	52.3
NCYL	*σ_max_* (MPa)	428	634	632	E (GPa)	34.8	52.7	52.3
BSAM/MIC	*σ_max_* (MPa)	485	581	551	E (GPa)	35.2	52.8	52.4
EHM	*σ_max_* (MPa)	425	605	602	E (GPa)	34.5	52.8	52.2

**Table 6:** Open hole tension results.

		Open Hole Tension Static Strength			Open Hole Tension Stiffness			
Team	Static Strength	[30, 60, 90, −60, −30]_2S_	[0, 45, 90, −45]_2S_	[60, 0, −60]_3S_	Elastic Modulus	[30, 60, 90, −60, −30]_2S_	[0, 45, 90, −45]_2S_	[60, 0, −60]_3S_
Experiment	*σ_max_* (MPa)	409	554	543	E (GPa)	32	48	49
MMF	*σ_max_* (MPa)	402	529	466	E (GPa)	32.4	48.3	48.8
GENOA	*σ_max_* (MPa)	405	543	435	E (GPa)	38.6	56.4	57.2
Helius PFA	*σ_max_* (MPa)	400	524	485	E (GPa)	35	50.6	50.9
DCN	*σ_max_* (MPa)	386	458	428	E (GPa)	35.2	50.9	51.2
MDS-C	*σ_max_* (MPa)	425	550	558	E (GPa)	34.7	51.8	51
MAC/GMC	*σ_max_* (MPa)	378	464	433	E (GPa)	33.7	49.1	48.9
EST	*σ_max_* (MPa)	409	528	489	E (GPa)	35.4	51.1	51.1
NCYL	*σ_max_* (MPa)	373	557	502	E (GPa)	34.5	50.3	50.9
BSAM/MIC	*σ_max_* (MPa)	388	553	551	E (GPa)	34.1	48.7	49.3
EHM	*σ_max_* (MPa)	449	558	502	E (GPa)	34.6	50.8	50.8

**Table 7:** Open hole compression results.

		Open Hole Compression Static Strength			Open Hole Compression Stiffness			
Team	Static Strength	[30, 60, 90, −60, −30]_2S_	[0, 45, 90, −45]_2S_	[60, 0, −60]_3S_	Elastic Modulus	[30, 60, 90, −60, −30]_2S_	[0, 45, 90, −45]_2S_	[60, 0, −60]_3S_
Experiment	*σ_max_* (MPa)	295	341	358	E (GPa)	30	44	44
MMF	*σ_max_* (MPa)	308	366	388	E (GPa)	32.3	46.3	46.8
GENOA	*σ_max_* (MPa)	323	363	380	E (GPa)	27.4	38.4	39.1
Helius PFA	*σ_max_* (MPa)	283	308	299	E (GPa)	30.5	43.4	43.6
DCN	*σ_max_* (MPa)	297	329	296	E (GPa)	36.1	52.2	52.5
MDS-C	*σ_max_* (MPa)	271	317	352	E (GPa)	34.7	34.1	45.3
MAC/GMC	*σ_max_* (MPa)	330	368	320	E (GPa)	29.2	41.6	41.9
EST	*σ_max_* (MPa)	296	347	331	E (GPa)	30.9	43.9	43.7
NCYL	*σ_max_* (MPa)	304	341	299	E (GPa)	29.8	41.9	41.9
BSAM/MIC	*σ_max_* (MPa)	336	380	388	E (GPa)	29.6	41.7	41.9
EHM	*σ_max_* (MPa)	360	393	368	E (GPa)	31.8	45.2	44.8

## Data Availability

The data presented in this study are available on request from the corresponding author.
